# Noninvasive Electroencephalography Equipment for Assistive, Adaptive, and Rehabilitative Brain–Computer Interfaces: A Systematic Literature Review

**DOI:** 10.3390/s21144754

**Published:** 2021-07-12

**Authors:** Nuraini Jamil, Abdelkader Nasreddine Belkacem, Sofia Ouhbi, Abderrahmane Lakas

**Affiliations:** 1 Department of Computer Science and Software Engineering, College of Information Technology, United Arab Emirates University, Al Ain P.O. Box 15551, United Arab Emirates; 201990167@uaeu.ac.ae (N.J.); sofia.ouhbi@uaeu.ac.ae (S.O.); 2 Department of Computer and Network Engineering, College of Information Technology, United Arab Emirates University, Al Ain P.O. Box 15551, United Arab Emirates; alakas@uaeu.ac.ae

**Keywords:** adaptive technology, assistive technology, brain–computer interface, EEG equipment, rehabilitative technology

## Abstract

Humans interact with computers through various devices. Such interactions may not require any physical movement, thus aiding people with severe motor disabilities in communicating with external devices. The brain–computer interface (BCI) has turned into a field involving new elements for assistive and rehabilitative technologies. This systematic literature review (SLR) aims to help BCI investigator and investors to decide which devices to select or which studies to support based on the current market examination. This examination of noninvasive EEG devices is based on published BCI studies in different research areas. In this SLR, the research area of noninvasive BCIs using electroencephalography (EEG) was analyzed by examining the types of equipment used for assistive, adaptive, and rehabilitative BCIs. For this SLR, candidate studies were selected from the IEEE digital library, PubMed, Scopus, and ScienceDirect. The inclusion criteria (IC) were limited to studies focusing on applications and devices of the BCI technology. The data used herein were selected using IC and exclusion criteria to ensure quality assessment. The selected articles were divided into four main research areas: education, engineering, entertainment, and medicine. Overall, 238 papers were selected based on IC. Moreover, 28 companies were identified that developed wired and wireless equipment as means of BCI assistive technology. The findings of this review indicate that the implications of using BCIs for assistive, adaptive, and rehabilitative technologies are encouraging for people with severe motor disabilities and healthy people. With an increasing number of healthy people using BCIs, other research areas, such as the motivation of players when participating in games or the security of soldiers when observing certain areas, can be studied and collaborated using the BCI technology. However, such BCI systems must be simple (wearable), convenient (sensor fabrics and self-adjusting abilities), and inexpensive.

## 1. Introduction

Most people know someone or have heard of someone who suffers from paralysis. In most cases, people become paralyzed because of accidents or medical conditions that partially or entirely affect the way their muscles and nerves function. People with disabilities do not usually receive the support they need because caring for them can be very expensive. Brain–computer interface (BCI) technology is now implemented in the treatment of patients suffering from physical impairments [1,2,3]. This technology promises to significantly enhance the quality of life of such patients by considerably improving their autonomy and mobility. Moreover, the idea of interfacing brains with machines has long captured human imagination [2]. Paralyzed people with neurological diseases (e.g., locked-in syndrome, muscular dystrophy, amyotrophic lateral sclerosis, brainstem stroke, and spinal cord injury) experience difficulties, such as in walking, speaking, and writing, because they lose fine motor control or lack a complete control of their voluntary muscles. However, their thinking capabilities are usually the same as those of nondisabled individuals. Generally, such patients are conscious and their mental abilities are unimpaired.

Consequently, research in neurology focuses on monitoring brain activities and incorporating the resulting data into BCIs. For instance, brain data can be used to allow patients to control home appliances or the movement of a wheelchair in four directions [4,5], send a message [6], or write an e-mail [6,7]. While Fazel-Rezai et al. [8] discussed some other applications that use the BCI technology. The brain activities can be noninvasively recorded using sensors on the scalp or invasively recorded using terminals set on the brain surface or inside the cerebrum. The popularity of such technologies is increasing because they can support the daily activities of people suffering from severe motor disabilities [9,10]. Healthy people can also use BCIs with other applications, particularly in games such as Tetris [11] and Brain Invaders, using the P300 paradigm with the OpenViBE platform [12]. They can also be used in virtual environments (aircraft simulators) [13].

**Figure 1:**
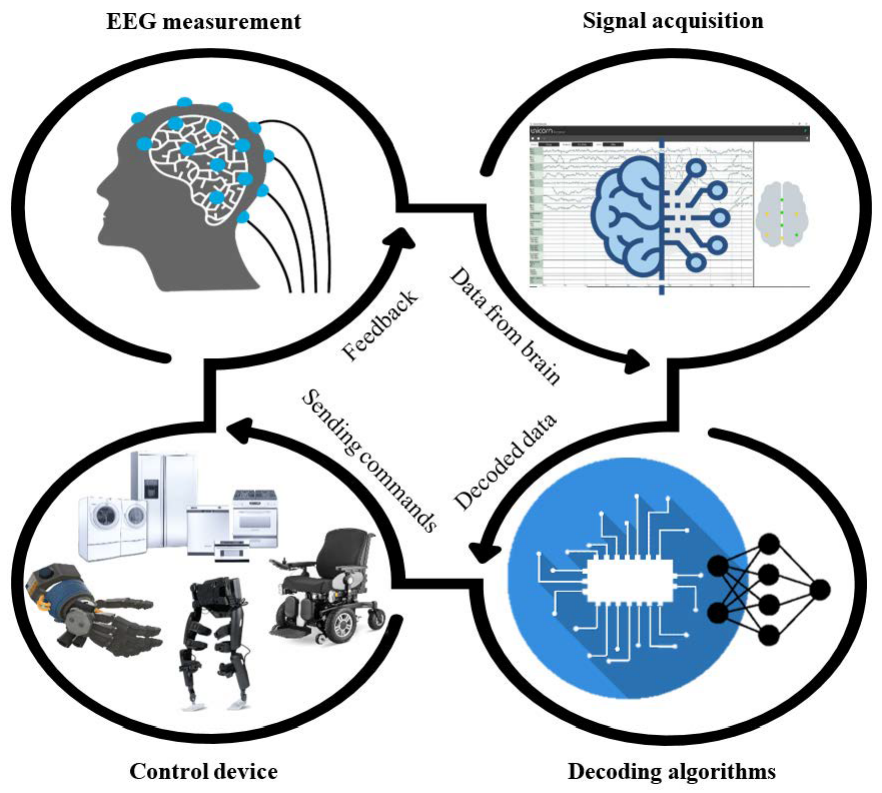
Brain–computer interface system.

BCI applications mainly aim to help people with severe motor impairments in living their lives like ordinary people as much as possible. Several devices using BCIs have been developed to assist in human activities [14]. Furthermore, several studies have proposed ways for helping people with severe impairments, such as paralysis and brain strokes, to ensure communication using direct links between brains and devices [4,15,16]. A BCI device’s main objective is to re-establish usable capacities of individuals with neuromuscular disorders. BCI procedures require the extraction of signal features from the cerebrum. Then, these features are analyzed and transformed into commands. An overview of a typical BCI system is illustrated in Figure 1.

The methods used to observe brain activities can be classified into noninvasive and invasive methods, and the contrast between these methods is based on the electrode placement. For invasive procedures, a patient must undergo neurosurgery, where an electrode is directly implemented into the brain using a single BCI unit or multiple units. The brain area can be monitored using the BCI unit [17]. Here, the signals produced are of high quality; however, the negative impact of this method is significant owing to the increase in the brain’s scar tissue [18]. An example of the invasive method is electrocorticography (ECoG), where brain activities are straightforwardly recorded from the brain surface [19].

**Figure 2:**
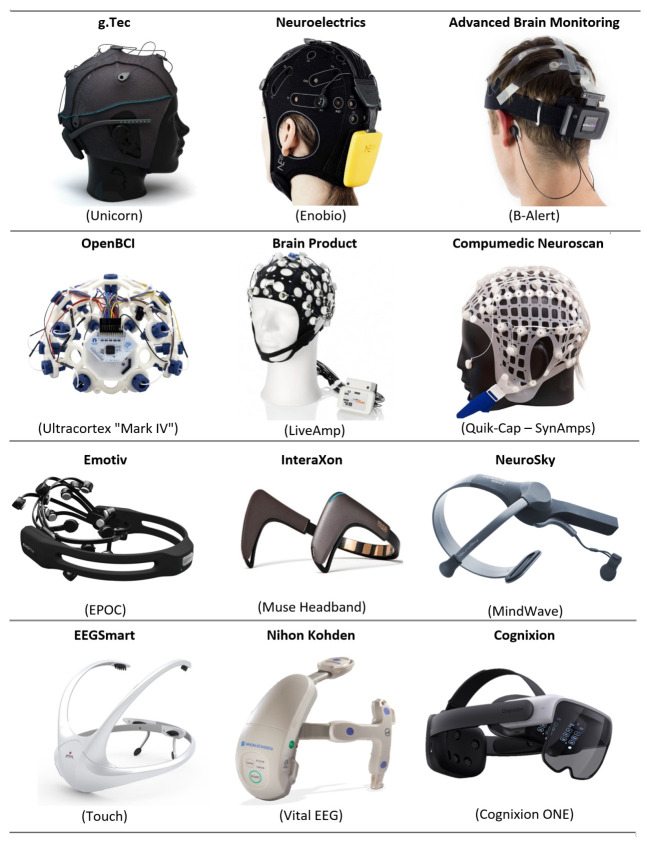
Examples of the commercial noninvasive EEG equipment based on the BCI technology. EEGSmart, Nihon Kohden, and Cognixion refer to the future noninvasive EEG designs.

The noninvasive method is less costly than the invasive method and causes minimal discomfort, and it is highly decisive [14]. For instance, an EEG tracks and records the brain wave patterns using electrodes placed on the scalp. Based on the acquired wave patterns, the signals are then dispatched to a computer. Figure 2 shows some commercial noninvasive EEG equipment.

The EEG primary paradigm includes motor imagery (MI), P300, and steady-state visual evoked potential (SSVEP) [20], which are different in the way in which they capture signals. These three paradigms have different approaches and potentials. In P300, if the subject is passionately engaged in a task, the EEG wave occurs with a high positive peak after 300 ms of a stimulus in a human-event-related potential. Conversely, MI is concerned with the psychological process of a movement without muscle actuation [21].

Rehabilitation is one of the therapy and training methods to restore the motor and muscle damage. Examples are robot-aided therapy [22] or using imagery cues to transform activity practices into daily routines suitable for therapy. For instance, a patient only imagines the action, such as grasping a bottle. Then, the brain signal sends the command to the device to control the movement. Remsik et al. [23] proposed two methods of using BCI systems for people with impairments to regain motor control. The first method is to train patients to deliver additional motor brain signals, and the second one is to train patients to activate devices that improve the motor function. The EEG method is revealing the remarkable improvements and continuous changes, even though individuals with procured motor impairments regularly display damaged cortices or problems with motor connections. Ramos-Murguialday et al. [24] introduced the aftereffects of the randomized control of a preliminary of 16 patients with chronic stroke using a BCI for the hand and arm orthotic feedback.

Assistive technology enables people with physical impairments to communicate, participate in communities, play, and move like ordinary people using a piece of equipment. This technology can reduce the stress of caregivers in terms of thinking about people with disabilities [25,26].

Adaptive technology refers to the enhanced versions of the existing tools that offer extra features and interaction opportunities to help people perform specific tasks [27], such as in education. Zhang [28] proved that students, particularly Chinese students, can better learn English using BCIs. Chiang et al. [29] measured the attention level of students and concluded that they can become more focused and learn better using BCIs.

To sum up, herein, a systematic literature review (SLR) is conducted to address the following research questions (RQs) as shown in Table 1.

Therefore, the main aim of this review is to identify the related literature with BCI equipment that can support various research fields. Further, the existing brands and types of commercial EEG equipment used for assistive, adaptive, and rehabilitative BCI technologies are reviewed.

This SLR is structured as follows. First, a research methodology was used to retrieve all the related articles by using online search databases (IEEEXplore, Scopus, PubMed, and ScienceDirect). Then, the analysis results from systematic review are summarized based on the company and type of devices. Finally, a discussion was performed, conclusions were drawn based on the findings, and future research areas were proposed to enhance the impact of the result.

## 2. Methods

### 2.1. Search Strategy

**Figure 3:**
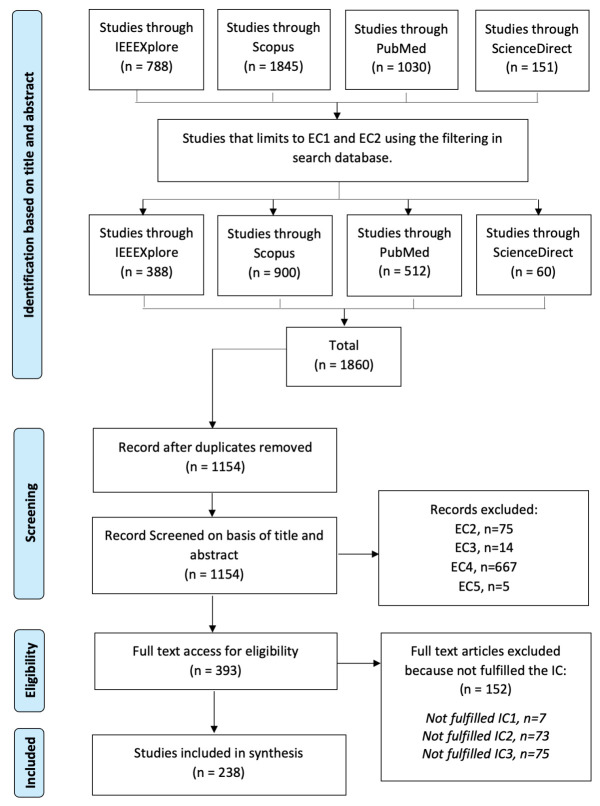
Flow process using the PRISMA method.

This SLR was investigated considering the rehabilitative, assistive, and adaptive technologies using the noninvasive BCI technology based on the preferred reporting items for systematic reviews meta-analyses (PRISMA) [30] (Figure 3). The following online electronic databases were used to search for relevant studies: PubMed, IEEE, Scopus, and ScienceDirect. The search string used in the search process was “(BCI OR “Brain–computer interface” OR BMI OR “brain–machine interface”) AND (EEG OR electroencephalogram) AND (rehab* OR assist* OR adapt*).” This search string was used in the digital libraries to search for the title, keywords, and abstract of candidate publications. The search was finalized in early February 2021.

### 2.2. Inclusion and Exclusion Criteria

The studies included in this SLR were selected based on the following inclusion criteria (IC): (IC1) studies in which EEG was used as a noninvasive BCI, (IC2) studies in which commercial brands and companies of EEG equipment were mentioned, and (IC3) studies focusing on devices and applications for rehabilitation, assistive, and adaptive environments in real life.

The exclusion criteria (EC) for this SLR are (EC1) publications published before 2016 and after 2020, (EC2) studies that are not prereview papers or survey/review papers and chapters, (EC3) non-English articles, (EC4) studies that did not focus on devices and focused more on the outcomes of algorithms, classifications, and brain areas, and (EC5) articles that cannot be retrieved as full articles.

### 2.3. Data Extraction

We extract the following characteristic:

1. Brand and company: The brand names of the EEG equipment and the company names that produce them.
2. Type of EEG equipment: The articles that use either wired or wireless equipment. Nowadays, many applications provide wireless equipment because of its low cost and physical mobility [31].
3. The sector of using EEG equipment: A broad area of applications that use BCIs as information sources [32].

## 3. Results

A considerable number of papers were analyzed, including those concerned with assistive, adaptive, and rehabilitative BCI technologies. This SLR retrieved 1860 articles; however, only 238 articles were included after screening using the aforementioned eligibility criteria. EC2 was employed again because some databases did not provide specific research type filters (Figure 3). Further, some reviews and survey papers are often identified as journal types.

**RQ1: What are the publication trends based on EEG equipment?**

**Figure 4:**
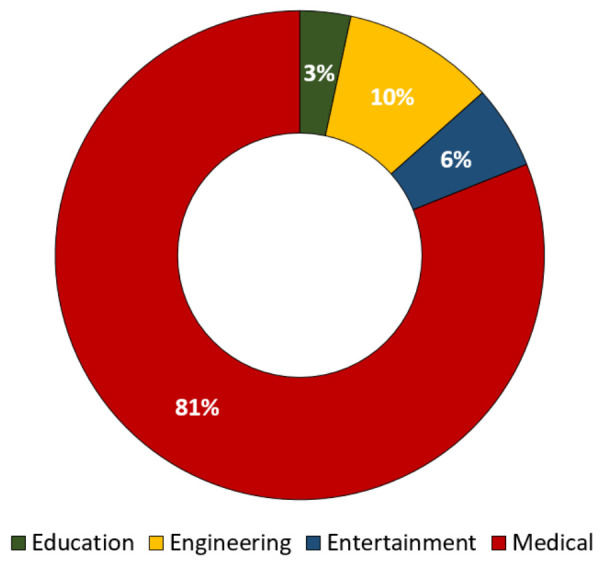
Proportion chart of published article research areas based on noninvasive BCI technology.

Figure 4 shows the percentage proportion chart of the four main research areas identified in this SLR: education, engineering, entertainment, and medicine. Overall, 81% of the articles discussed BCI in medicine areas because rehabilitation is synonymous with health and medical care. While the engineering area covered approximately 10% of the total articles, some articles proposed BCI systems to assist while drive the vehicles [33]. The engineering area here does not focus on medical purposes such as on controlling the drone or vehicles [34]. Further, 6% and 3% of the articles were based on the entertainment and education areas [35], respectively.

**Figure 5:**
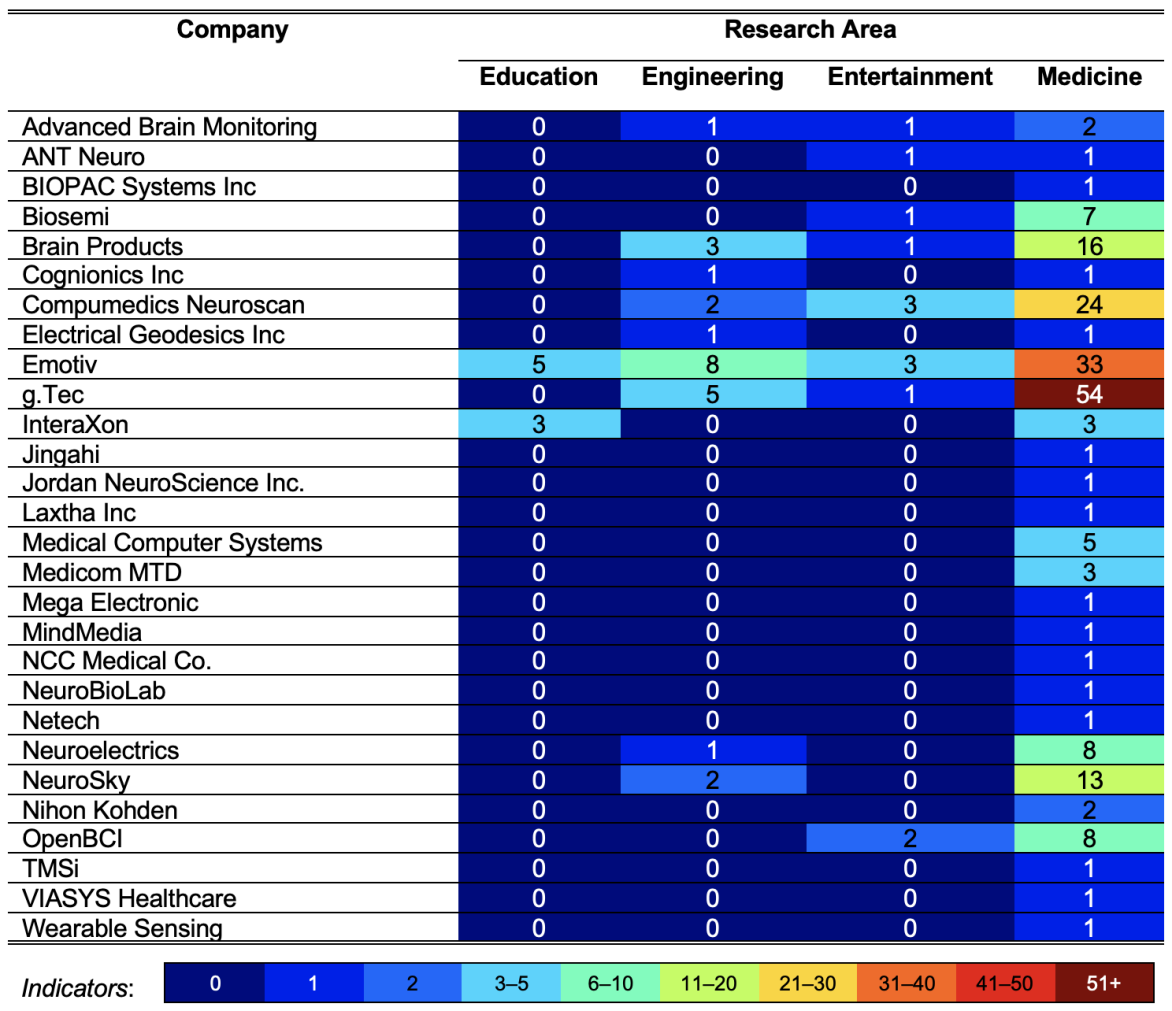
Mapping between the companies and research areas identified in the selected articles. Different colors represent the proportions of the reviewed studies, as shown by the indicators.

A total of 28 companies were identified from the selected studies in this SLR. Figure 5 presents information on the EEG equipment in the selected studies and their companies. According to Figure 5, the g.Tec company leads with 60 articles. One of the reasons many studies used the equipment of g.Tec is that this company has four headquarters in Austria, Spain, USA, and Hong Kong (https://www.gtec.at/, accessed on 25 February 2021) which can be easily obtained and purchased and at the same time can match the power consumption in different countries. The equipment from Emotiv company was used in 49 articles and that from Compumedics Neuroscan company was used in 29 articles. The equipment of Brain Products, NeuroSky, and OpenBCI was used in 20, 15, and 10 articles, respectively. The equipment of the rest of the companies was less, i.e., 10 articles.

The details of each research area and the equipment from different companies are:

*Education*: Only eight articles were found in this area with two companies. Five articles used Emotiv equipment [36,37,38,39,40] and another three articles used the equipment from InteraXon company [41,42,43].

*Engineering*: This is the second highest research area in this SLR, with 24 articles and 9 companies. Eight articles used Emotiv equipment [5,44,45,46,47,48,49,50], five articles used g.Tec equipment [51,52,53,54,55], three articles used Brain Products equipment [56,57,58], two articles used Compumedics Neuroscan equipment [59,60], and two articles used NeuroSky equipment [61,62]. For the equipment for Cognionics Inc. [63], Electrical Geodesics Inc. [64], Neuroelectrics [65], and Advanced Brain Monitoring [66], one article each used their equipment.

*Entertainment*: Only 13 articles were discussed in this area. Compumedics Neuroscan [67,68,69], and Emotiv [70,71,72] equipment was employed in three articles each. Two articles used OpenBCI equipment [73,74] and one article each used the equipment of Biosemi [75], g.Tec [76], ANT Neuro [77], Brain Products [78], and Advance Brain Monitoring [79].

*Medical*: This is the most popular research area in this SLR, with 193 articles. g.Tec equipment was used in 54 articles for the EEG method [80,81,82,83,84,85,86,87,88,89,90,91,92,93,94,95,96,97,98,99,100,101,102,103,104,105,106,107,108,109,110,111,112,113,114,115,116,117,118,119,120,121,122,123,124,125,126,127,128,129,130,131,132,133], 33 articles used Emotiv equipment [134,135,136,137,138,139,140,141,142,143,144,145,146,147,148,149,150,151,152,153,154,155,156,157,158,159,160,161,162,163,164,165,166], and 24 articles used Compumedics Neuroscan equipment [167,168,169,170,171,172,173,174,175,176,177,178,179,180,181,182,183,184,185,186,187,188,189,190]. Further, 16 articles used Brain Products equipment [191,192,193,194,195,196,197,198,199,200,201,202,203,204,205,206], 13 articles used NeuroSky equipment [207,208,209,210,211,212,213,214,215,216,217,218,219], and Neuroelectrics [220,221,222,223,224,225,226,227] and OpenBCI [228,229,230,231,232,233,234,235] equipment were used in eight articles each. Moreover, seven articles used Biosemi equipment [236,237,238,239,240,241,242], and four articles used Medical Computer Systems equipment [243,244,245,246]. Three articles used Medicom MTD equipment [247,248,249], and another three articles used the equipment from InteraXon company [250,251,252]. Two articles each used Advanced Brain Monitoring [253,254] and Nihon Kohden [255,256] equipment. One article each used the equipment of Mega Electronic [257], Jordan NeuroScience Inc. [258], BIOPAC Systems Inc. [259], Laxtha Inc. [260], TMSi [261], NeuroBioLab [262], Cognionics Inc. [263], Jingahi [264], VIASYS Healthcare [265], ANT Neuro [266], Wearable Sensing [267], Netech [268], MindMedia [269], NCC Medical Co. [270], and Electrical Geodesics Inc. [271].

**RQ2: What are the most common types (wired or wireless) of noninvasive EEG-based BCI equipment that have been used in brain studies?**

**Figure 6:**
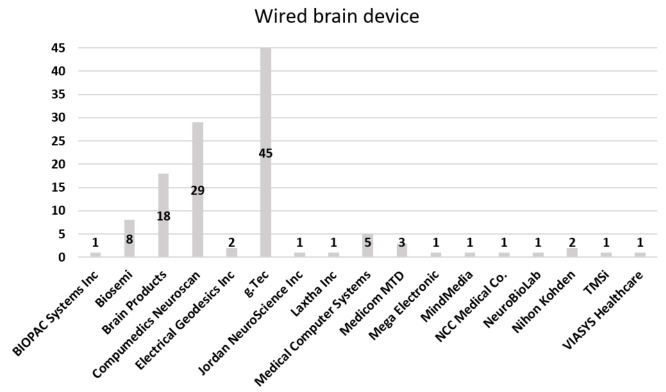
Proportion of companies that used noninvasive-wired equipment.

**Figure 7:**
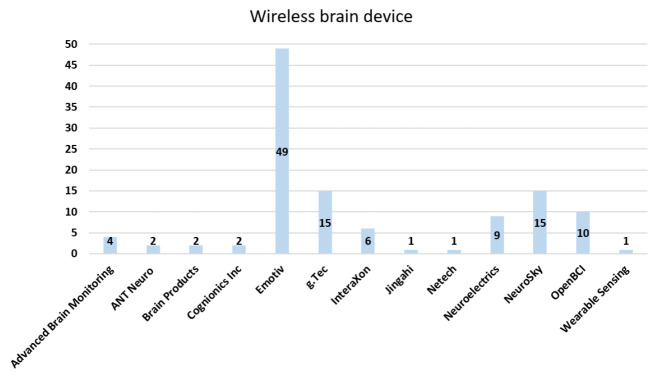
Proportion chart of companies that used noninvasive wireless equipment.

Figure 6 illustrates the proportion chart of wired EEG equipment. Overall, 45 articles used the wired equipment from the g.Tec company, followed by Compumedics Neuroscan and Brain Products, with 29 and 18 articles, respectively. The fourth popular brand of wired equipment was Biosemi, with eight articles. Figure 7 illustrates the proportion chart of wireless EEG equipment. Most articles used the wireless equipment of Emotiv, with 49 articles. The second highest were g.Tec and NeuroSky, each with 15 articles. Only two companies provided both types of EEG equipment, namely, Brain Products and g.Tec. The rest of the companies either provided wired or wireless equipment for BCI, and the devices could communicate with the brain using either wired or wireless models. The wired model is a standard system, whereas the wireless model is an evolving solution. Many companies have designed portable headsets to provide greater comfort at a low cost [272].

Table 2 summarizes the companies and their equipment, which were used in specific research areas. In Table 2, the SLR is divided into three main categories: company name (n = 28), the type of EEG equipment (wired or wireless), and research area (education, engineering, entertainment, and medicine). Furthermore, only the wireless equipment from Emotiv company covered all four research areas, even though the sector of Emotiv mostly offers entertainment options and performance (http://bnci-horizon-2020.eu/images/bncih2020/FBNCI_Roadmap.pdf, accessed on 25 February 2021).

Overall, 17 companies provided more than two brands/products with different characteristics. For example, the company Neuroelectrics offers Enobio 8 and Enobia 32, which have up to 8 and 32 channels for EEG recording, respectively (https://www.neuroelectrics.com, accessed on 25 February 2021). Therefore, researchers can select the necessary equipment based on their specific goals, including user acceptance, usability, and performance. The education research area used the wireless equipment from two companies: Emotiv (Emotiv EPOC and Emotiv Insight) and InteraXon (Muse headband). While Augustian et al. [64] used the equipment of Electrical Geodesics Inc. to propose applications and devices for assisting forestry crane control.

Table 3 shows the comparison between EEG equipment that is used in the selected studies. The details of the information about EEG equipment were found through the company’s website and some from the company’s representative. Unfortunately, three pieces of EEG equipment are no longer available, which are Refa32 (TMSi) and QuickAmp USB and V-amp (Brain Products). Soufineyestani et al. [273] made the comparison and limitation of EEG sensors available in the market specially for engineers, scientists, and clinicians, to understand more about EEG equipment that can match their preference. The symbol “-” implies that it could not uncover the information for the price. Some of the equipment have to request a quotation. Additionally, the symbol “X” in the Medical license represents that the company does not have the certificate for medical use. In column “Additional sensor”, the “X” indicates the equipment is without an additional sensor. There is a wide range of companies in the market with very different goals, driven by new applications and approaches. Each of the companies have specific goals to produce the BCI-based system. Advanced Brain Monitoring develops tools for alertness and monitoring sleep, and Emotiv focuses on the gaming and research market. Different companies target the other market sector such as Brain Products and Biosemi for research, g.Tec more on health and neurofeedback, assistive technology, and research. While Emotiv pivots on entertainment and performance (http://bnci-horizon-2020.eu/images/bncih2020/FBNCI_Roadmap.pdf, accessed on 25 February 2021). Based on the gathered information, each company has approximate applications for each EEG equipment in column “Recommendation” as shown in Table 3.

## 4. Discussion

Several researchers are currently studying BCI technologies in different domains, such as medicine, entertainment, finance, education, and wellness. Many of the studies included in this SLR covered the medicine sector because BCIs are commonly used in assistive technologies, particularly for people with severe disabilities [121,189,274] with regard to rehabilitation and treatment. This observation is not surprising because BCIs are commonly viewed as prospects for enhancing the lives of innumerable disabled people using assistive technologies. For instance, the wearable knee exoskeleton device is used to strengthen and rehabilitate the gait and restore the movement of people with knee motion disabilities [275]. Another application is controlling wheelchairs so that paralyzed or quadriplegic patients can move around without the need for caregivers [37,214,215].

Moreover, in the field of engineering, BCIs can provide disabled and healthy people with an alternative communication medium that involves minimum movement of muscles for activities such as controlling home appliances. Even healthy people may face circumstances where they cannot use their hands to operate appliances, e.g., during cooking or dealing with hazardous materials or chemicals. Therefore, hands-free systems have been established to control home appliances [48] or control robots that can help perform house chores, such as cleaning [5]. Currently, BCI-based home appliances can only handle one application at a time, thus limiting their effectiveness in real-world circumstances. Future research is needed to overcome this issue.

The prospect of BCI is applicable to both indoor and outdoor applications. The outdoor applications include helping security teams explore opponent areas for safety and security [50,276]. Other innovations using the BCI technology influence researchers to use BCIs as a part of daily life, such as controlling vehicles for assistance in remote driving [49]. With this approach, BCIs can help in programming vehicles for self driving whenever the vehicle detects that the driver feels drowsy or tired.

Nevertheless, intense attention is now centered on assistive applications meant to support healthy users, for example, in the entertainment and gaming areas. With some music, such applications can stir feelings and generate various emotional responses because music itself is a central feature of media-based entertainment [277]. Therefore, using BCI technology, music can be successfully produced based on emotion-based qualities using EEG data. However, there are some challenges when producing the same music for different people. For example, two people in one room may feel excited when listening to the same music; however, there is the possibility that one of them may not like the music. Hence, personal preferences also affect this sector.

Additionally, research on the use of BCI for gaming is increasing in the entertainment industry. Laar et al. [278] discussed the performance and experience of gaming using BCI equipment. Nevertheless, BCIs are radically different from other input devices, such as keyboards and mice, as they offer users alternative input channels for controlling games. Moreover, BCIs can assist players in being more engaged with games [71]. Some companies have established BCI games, and some BCI companies engage in game production, such as NeuroSky, Emotiv, and MindGames. Furthermore, games can help in rehabilitation and people can enjoy them and feel entertained. Vega et al. [70] proposed a game for controlling video games using prosthetic devices and Rashid et al. [61] proposed a game for increased attention, particularly for people with attention deficit disorder. However, the challenge in implementing BCIs in gaming can be the user experience. Wearing another device on the head can be uncomfortable for some users who play games for a long duration.

Further, BCIs can be tools for students to increase their learning ability and for educators to further understand their students. A BCI can analyze a student’s cognitive states, assess the information and visualizations to be used, and change the training method to respond to a student’s individual learning needs [38]. Furthermore, a BCI can help students refocus and increase their interest and engagement in the learning process [40]. The neurofeedback system in BCIs can help teachers to better observe and understand students, and it can aid them in customizing their approach based on a student’s needs. The Unicorn Hybrid Black brand of the g.Tec company was specifically designed for the education area. However, none of the articles discussed here used the Unicorn Hybrid Black equipment in the education area. The issues regarding BCI and education are mainly concerned with the costs of providing all students with the necessary equipment and collecting their EEG data and disturbances attributed to external variables, such as shifting students’ attention and noise in the class environment.

Furthermore, when the various industries’ potential expands, like entertainment and education, many companies join the market with very different objectives influenced by the emerging market sectors and approaches. Not all companies design conventional BCI systems; however, investments in consumer goods would undoubtedly and significantly affect the infrastructure, price, and usability for the BCI systems (http://bnci-horizon-2020.eu/images/bncih2020/FBNCI_Roadmap.pdf, accessed on 25 February 2021). Some companies provide both wired and wireless systems, and some companies only focus on developing and designing wireless equipment, such as InteraXon, Emotiv, and Neuroelectrics. Over the decades, the EEG hardware technology has also advanced. Many wireless multichannel devices have been developed, offering high-quality EEG and physiological signals in more straightforward, compact, and comfortable configurations compared with conventional complicated systems. Wireless systems are more convenient for users because they facilitate free movement. Moreover, wireless equipment has proven to be suitable for clinical trials [279]. However, some challenges need to be addressed regarding EEG equipment in sensor development. Not many researchers are deeply engaged in studying the BCI sensor design because it is generally a very complicated subject [280]. Sensors are applied to both wired and wireless equipment because they are attached to the human head. The electrode position must be accurate. Furthermore, the sensor material must be selected appropriately to avoid any side effect.

## 5. Limitation

This SLR may have several limitations that can impact its overall quality

- Other digital libraries could have been used in the search for studies, which may impact candidate studies. However, we selected the largest libraries that deal with the SLR’s topic. Using other libraries may have just resulted in more duplicates.
- Herein, the number of selected studies can impact the conclusion drawn. The studies are pertaining to the medicine sector because of the keyword “rehab*”. However, we included two other keywords related to other sectors in the search string.
- This review mainly focused on English publications; however, there could be relevant publications in other languages.

## 6. Conclusions

Assistive technology can benefit people with cognitive problems and physical impairments. In this review, a variety of equipment that can be successfully implemented using BCIs in four sectors (i.e., education, engineering, entertainment, and medicine) is highlighted. Overall, the EEG channel is a reference for adapting the technology based on different levels of impairments and disabilities. Moreover, rehabilitation can help people increase their independence using BCIs.

Based on the obtained results, numerous studies were biased toward the medicine sector. However, with further research and experiments, this trend can also be observed for the other sectors. EEG data could be efficiently obtained from all four research areas. Moreover, EEG devices can be used by healthy people, not only by people with disabilities. With an increasing number of wireless devices, EEG can be recorded in many natural ways in daily life, such as in crowded places or during exercise.

We do believe that using relatively expensive EEG devices that have FDA certificate in developing medical BCI applications is mandatory rather than using cheap portable devices due to their high quality signals in terms of signal-noise ratio (SNR) and spatio-temporal resolutions. For developing noninvasive BCI applications, investigators can refer to market size to decide which research studies to support. Nowadays, the BCI market is large and popular in terms of gaming applications. The manufacturer of the game console and some game developers can integrate with a BCI-based gaming device. For BCI technology to succeed and gain wide adoption, affordability should be guaranteed and focus on specific end-user products based on market demand for BCI investors (researchers) to decide which devices to select for the application. Wireless devices are more comfortable to move around and suitable for long-term use in daily life and outdoor applications. Additional, dry EEG electrode is easy to use and does not require additional instruments like syringes and there is no need to wash the head afterward.

Furthermore, a limited number of studies have reported using BCI in the engineering area, suggesting more room for exploration. With COVID-19, using EEG-based robot or drone control can explore and detect the location of the virus. The rationale is to reduce the risk of exposure of COVID-19 to the people. Next, in the entertainment sector, rather than focusing only on games, EEG-based BCI can predict the market for the movie genres (e.g., which genre can give more excitement to the audience). In the same way, portable EEG devices are important in neuro-education. Due to COVID-19, all the schools and institutes were closed and were instructed to perform distance learning. When studying online, the optimal learning strategy can be determined by analyzing the behavior of student brains.

The BCI-based system can expand into the new market, which gives more significant opportunities by merging the current market with another field, such as medicine, with the robotic sector to reduce overall healthcare costs. Due to EEG devices’ widespread availability and becoming portable, the treatment and rehabilitation are not limited to the hospital but may also occur in the home. Another instance is engineering with the automotive and aerospace sectors for people’s safety in the road or outer space. Diverse considerations should be made before choosing and purchasing a suitable device.

Numerous factors need to be considered for selecting the devices such as medical certificate, electrode type (dry/saline/gel), size and shape for the cap, and type of devices (wired/wireless). The applications specific to medical and clinical treatment need approval. Kasim et al. [281] mentioned the dry electrode exhibited a greater resistance to line noise. Using several different sizes and shapes (cap) for head size variability experiments and studies can increase the overall price because of the need to buy more than one cap. Li et al. [282] mentioned that the wired EEG devices give better quality but are more expensive, while wireless devices are more convenient for daily life application [283]. The wireless device was suited for underlying cognitive processes and body motion such as sport science and physical therapy.

More user-experience evaluations and data-integrity policies are required to ensure that the needs and preferences of the end users are met and that their personal data are secured. Therefore, the obtained findings can impact policymakers with regard to using EEG devices, particularly wireless devices, to prevent data from being stolen and for researchers to explore new sectors to adapt the BCI technology.

Moreover, SLR results provide directions for future research. In future studies, we intend to measure the user experience based on different devices to identify the extra features of each EEG equipment and determine which of them is suitable for each sector.

## Figures and Tables

**Table 1:** SLR research questions.

	Research Question	Rationale
**RQ1**	What are the publication trends based on EEG equipment?	To identify the sector that collaborates with BCI technology using the EEG method
**RQ2**	What are the most common types (wired or wireless) of noninvasive EEG-based BCI equipment that have been used in brain studies?	To discover whether the EEG equipment mostly uses the wired or wireless type based on a specific research area

**Table 2:** Types of EEG equipment for each company.

Company	Type of EEG Equipment		Research Area
	Wired	Wireless		
Advanced Brain Monitoring	×	B-Alert^®^ X10	EG [66], MD [253,254]
		B-Alert X24	ET [79]
ANT Neuro	×	eegosports	ET [77]
		eego™rt	MD [266]
BIOPAC Systems Inc.	EEG100C	×	MD [259]
Biosemi	ActiveTwo	×	ET [75], MD [236,237,238,239,240,241,242]
Brain Products	actiCAP system		MD [194,199,205]
	actiHamp		MD [192,198,201,203]
	BrainAmp		EG [57], ET [78], MD [195,196,197,200,204]
	BrainVision		EG [58]
	capTrak		MD [206]
	QuickAmp USB		EG [56]
		V-amp	MD [191]
		MOVE system	MD [193]
Cognionics Inc.	×	HD-72 EEG	EG [63]
		Quick-20	MD [263]
Compumedics Neuroscan	Grael	×	ET [67,68], MD [187,188]
	NuAmps		MD [167,168,169,170,172,176,180,183,185,186,189,190]
	SynAmps		EG [59,60], ET [69], MD [171,173,174,175,177,178,179,181,182,184]
Electrical Geodesics Inc.	Geodesic EEG System 400	×	EG [64], MD [271]
Emotiv	×	Emotiv EPOC	ED [37,39,40], EG [5,44,45,46,47,48,49,50], ET [70,71,72], MD [134,135,136,137,138,139,140,141,142,143,144,145,146,147,148,149,150,151,152,153,154,155,156,157,158,159,160,162,164,165,166]
		Emotiv Insight	ED [36,38], MD [161,163]
g.Tec	g.BSamp		MD [110]
	g.Hiamp		MD [85,97,100,106,127,132]
	g.USBamp		EG [52,54], ET [76], MD [80,81,82,83,84,87,89,92,94,95,96,98,99,101,102,104,105,107,108,111,112,114,117,118,119,120,121,122,125,126,128,130,131,133]
		g.MOBIlab+^®^	MD [86,93,103,113,115,129]
		g.Nautilus	EG [51], MD [88,90,91,109,116,123,124]
		Unicorn Hybrid Black	EG [55]
InteraXon	×	Muse headband	ED [41,42,43], MD [250,251,252]
Jingahi	×	JAGA16	MD [264]
Jordan NeuroScience Inc.	BrainNet	×	MD [258]
Laxtha Inc.	PolyG-I	×	MD [260]
Medical Computer Systems	NVX52	×	MD [243,244,245,246]
Medicom MTD	Encephalan-EEGR-19/26	×	MD [247,248,249]
Mega Electronic	NeurOne	×	MD [257]
MindMedia	Nexus10 Biosignal	×	MD [269]
NCC Medical Co.,	NGERP-P	×	MD [270]
NeuroBioLab	NBL640	×	MD [262]
Netech	×	MinSim300	MD [268]
Neuroelectrics	×	Enobio 8	EG [65], MD [220,223,224,226]
		Enobio 32	MD [227]
		StarSim 8	MD [222]
		StarSim R32	MD [221,225]
NeuroSky	×	BrainWave	MD [208,210]
		MindFlex	MD [219]
		MindWave Mobile	EG [61,62], MD [207,209,211,212,213,214,216,217,218]
		ThinkGear AM (TGAM)	MD [215]
Nihon Kohden	JE-921A	×	MD [256]
	AB-611J		MD [255]
OpenBCI	×	OpenBCI 32 bit	MD [232,235]
		Open BCI Cyton	ET [73,74], MD [228,229,231,234]
		OpenBCI Ganglion	MD [233]
		Ultracortex BCI	MD [230]
TMSi	Refa 32	×	MD [261]
VIASYS Healthcare	Nicolet 1	×	MD [265]
Wearable Sensing	×	DSI-24	MD [267]

**Table 3:** A systematic comparison of the EEG devices.

Company	EEG Equipment	Medical Certificate	Recommendation	Electrode Number, Type and Placement	Additional Sensor (Optional)	Size and Shape (Cap)	Approximated 2021 Cost	No. of Publication
Advanced Brain Monitoring	B-Alert^®^ X10	ISO 13485, CE, FDA	Neuromarketing, BCI, identify biomarkers	9 channels, electrolyte cream, and cover whole brain	ECG, EMG, EOG	Adjustable (from adolescent to adult)	$9950 up to $14,950	4
	B-Alert X24			20 channels, electrolyte cream, and cover whole brain				
ANT Neuro	eegosports eego™rt	CE, FDA	BCI, neurofeedback, neurorehabilitation, neurogaming	8 to 64 channels, gel/soft dry and cover whole brain	EMG, physiological sensor	6 sizes	-	2
BIOPAC Systems Inc.	EEG100C	X	Epilepsy, tumor pathology, sleep studies, evoked responses, cognition studies.	16 channels, wet and cover whole brain	X	4 sizes	$2000	1
Biosemi	ActiveTwo	X	Electrophysiology research	16 to 256 channels, gel and cover whole brain	EMG, ECG	15 sizes	17,000 up to 75,000	8
Brain Products	actiCAP system actiHamp BrainAmp BrainVision capTrak MOVE system	X	Neuroscience, neurofeedback, neurophysiological	8 to 256 channels, gel/dry and cover whole brain	EOG, EMG	14 sizes	$12,000 to $28,500	20
	QuickAmp USB V-amp	No longer available						
Cognionics Inc.	HD-72 EEG	X	Neurofeedback, neurodiagnostic	64 channels, dry and cover whole brain	ECG, EMG, EOG, RESP, GSR	Adjustable	$14,500 up to $26,000	2
	Quick-20			21 channels, dry electrode and whole brain				
Compumedics Neuroscan	Grael NuAmps SynAmps	FDA	Clinical neuro-diagnostics, research	up to 256 channels, gel/saline and cover whole brain	EOG, ECG, EMG	5 sizes	-	29
Electrical Geodesics Inc.	Geodesic EEG System 400	FDA	Clinical applications	up to 256 channels, saline and cover whole brain	ECG	Available in sizes from infant to adult	-	2
Emotiv	Emotiv EPOC	X	Research, personal use	14 channels, saline soaked felt pads and cover whole brain	Quaternions, accelerometer, magnetometer	Adjustable	$299 up to $849	49
	Emotiv Insight			5 channels, semi dry and cover frontal, temporal and parietal				
g.Tec	g.BSamp g.Hiamp g.USBamp g.MOBIlab+^®^ g.Nautilus Unicorn Hybrid Black	ISO 14971, FDA	BCI, neuroscience, neurotechnology	up to 256 channels, dry/gel and cover whole brain	ECoG, ECG, EMG, EOG, accelerometer, external body sensor	3 sizes	1000(Unicorn Hybrid Black) to 30,000(customize)	60
Jingahi	JAGA16	X	Neuroscience, suitable for rat	16 channels and cover whole brain	X	Standard size	-	1
Jordan NeuroScience Inc.	BrainNet	FDA	Neurodiagnostic, neurofeedback	14 to 21 channels, cream and cover whole brain	X	4 sizes	-	1
Laxtha Inc.	PolyG-I	ISO 13485, KFDA	Scientific research, forensic science	8 channels and prefrontal area	ECG, EMG, PPG, GSR, RESP	Standard size	-	1
Medical Computer Systems	NVX52	ISO 13485	Research for any application	48 channels, wet and cover whole brain	Any biosensor	9 sizes	4860	5
Medicom MTD	Encephalan-EEGR-19/26	ISO 13485	Neurology, neurophysiology, epileptology, sleep studies, scientific research	20 channels, wet and cover whole brain	EOG, ECG, EMG	5 sizes	$5000 up to $40,000	3
Mega Electronic	NeurOne	European Medical Directive 93/42/EEC, ISO 13485, CE	Neuroscience, psychological aplication	32 to 128 channels and cover whole brain	Gyro, EMG, GSR, accelerometer,	Standard size	-	1
MindMedia	Nexus10 Biosignal	CE, FDA	Biofeedback, neurofeedback, psychophysiological research.	4 channels, electrogel and cover whole brain	EMG, EOG, ECG	4 sizes	1050	1
Interaxon	Muse headband	X	EEG-powered sleep, tracking, meditation	2 channels, dry and cover frontal lobe	Gyro, PPG, accelerometer	Adjustable	$294.98 up to $369.98	6
NCC Medical Co.	NGERP-P	ISO 13485	-	24 channels and cover whole brain	-	Standard size	-	1
NeuroBioLab	NBL640	-	Neurobiofeedback	24 channels, dry/gel electrode and cover whole brain	X	Standard size	-	1
Netech	MinSim300	CE, FDA	Recorders, sleep study monitors	10 channels	-	Standard size	-	1
Neuroelectrics	Enobio 8 Enobio 32 StarSim 8 StarSim R32	CE, FDA	Neuroscience, BCI, neurogaming, neurofeedback	8 to 32 channels, dry/gel and cover whole brain	Accelerometer	6 sizes	-	9
NeuroSky	BrainWave MIndFlex MindWave ThinkGearAM (TGAM)	X	BCI, neurogaming, neurofeedback, neuroscience, meditation	1 channel, dry and cover frontal lobe	ECG	Adjustable	$109.99	15
Nihon Kohden	JE-921A AB-611J	ISO 13485, MDSAP	Epilepsy monitoring, medical research	5 to 32 channels and cover whole brain	Oximetry	4 sizes and 1 adjustable silicone cap	-	2
OpenBCI	OpenBCI 32bit Open BCI Cyton OpenBCI Ganglion Ultracortex BCI	X	BCI, biosensing, neurofeedback	4 to 21 channels, dry/gel and cover whole brain	EMG, ECG, accelerometer	3 sizes and 3D printable can be adjust	up to $3200	10
TMSi	Refa 32	No longer available						1
VIASYS Healthcare	Nicolet 1	FDA	Respiratory care, neuroscience, medical, surgical care	32 to 44 channels and cover whole brain	EMG	Standard size	-	1
Wearable Sensing	DSI-24	X	Psychological research, neuroscience, neuromarketing, BCI, neurogaming, neurofeedback	21 channels, dry and cover whole brain	ECG, EMG, EOG	Adjustable	20,000	1

ISO-International Organization for Standardization, CE-Conformitè Europëenne, FDA–Food and Drug Administration, KFDA-Korea MFDS (KFDA) Medical Device Registration and Approval, MDSAP-Medical Device Single Audit Program, ECG-Electrocardiogram, EMG-Electromyography, EOG-Electrooculography, ECoG-Electrocorticography, PPG-Photoplethysmography, GSR-Galvanic Skin Response, RESP-Respiratory. NB: Some EEG device prices could not be easily found from the company website. Therefore, we did not show the costs of all devices in the table.

## Data Availability

Data generated from the IEEE digital library, PubMed, Scopus, and ScienceDirect at UAEU, available upon request.
